# Generation of Induced Pluripotent Stem Cells from Patients with Multiple Myeloma

**DOI:** 10.4274/tjh.galenos.2021.2020.0682

**Published:** 2021-12-07

**Authors:** İrem Yılmaz Başaran, Erdal Karaöz

**Affiliations:** 1 Eskişehir Osmangazi University, Cellular Therapy and Stem Cell Production Application and Research Center, Eskişehir, Turkey; 2 İstinye University, Faculty of Medicine, Department of Histology and Embryology, İstanbul, Turkey; 3 İstinye University, Center for Stem Cell and Tissue Engineering Research and Practice, İstanbul, Turkey; 4 İstinye University, 3D Bioprinting Design and Prototyping R&D Center, İstanbul, Turkey; 5 Liv Hospital, Center for Regenerative Medicine and Stem Cell Manufacturing (LivMedCell), İstanbul, Turkey

**Keywords:** Induced pluripotent stem cells, Multiple myeloma, Mesenchymal stem cells, Sendai virus

## Abstract

**Objective:**

Patient-specific induced pluripotent stem cells (iPSCs) have potential in human disease modeling and regenerative medicine. The in vitro phenotype of disease-specific iPSC-derived cells can be used to bridge the knowledge gap between clinical phenotype and molecular or cellular pathophysiology and to understand the pathology of diseases, along with further applications, such as creating new strategies for drug screening or developing novel therapeutic agents. The aim of our study was to generate iPSCs from multiple myeloma (MM) patients.

**Materials and Methods:**

Mesenchymal stem cells (MSCs) isolated from MM patients were induced for pluripotency via the Sendai virus. Fibroblasts were used as a control. Microscopic analysis was performed daily. For colony selection, live staining was done using alkaline phosphatase staining. Reprogramming experiments were confirmed by flow cytometry, immunofluorescence (IF) staining, and gene expression analyses. To confirm the spontaneous differentiation potential, an in vitro embryonic body (EB) formation assay was performed.

**Results:**

Fibroblasts and MSCs obtained from MM patients were reprogrammed using the Sendai virus, which contains reprogramming vectors with the four Yamanaka factors, Oct3/4, Sox2, Klf4, and c-Myc. Microscopic analysis revealed that the generated iPSCs possessed classical embryonic stem cell-like morphological characteristics. Reprogramming experiments further showed that both cell lines can be reprogrammed up to the pluripotent stage, which was confirmed by flow cytometry, IF staining, and gene expression analyses. Spontaneous differentiation potential was confirmed by in vitro EB formation assays.

**Conclusion:**

iPSCs have been successfully obtained from MM patients for the first time. These cells could clarify the molecular mechanisms behind this disease.

## Abstract (tr)

**Amaç:**

Hastaya özgü uyarılmış pluripotent kök hücreler (uPKH) insan hastalık modellemesi ve rejeneratif tıpta büyük bir potansiyele sahiptir. Hastalığa özgü uPKH’den türetilen hücreler, klinik fenotip ile moleküler veya hücresel patofizyoloji arasındaki bilgi boşluğunu kapatmak ve ilaç taraması için yeni stratejiler oluşturmak ve yeni terapötik ajanlar geliştirmek gibi stratejilerle hastalık patolojilerini anlamada faydalı olacaktır. Çalışmamızın amacı multipl myelom (MM) hastalarından uPKH üretmektir.

**Gereç ve Yöntemler:**

MM hastalarından izole edilen MKH’ler Sendai virüs yoluyla uyarılarak pluripotensi aşamasına döndürülmüştür. Çalışmada fibroblastlar kontrol olarak kullanılmıştır. Her gün mikroskobik analiz yapılmış, koloni seçimi için alkalin fosfataz canlı boyaması yapılmıştır. Yeniden programlama deneyleri akış sitometrisi, immünofloresan (IF) boyama ve gen ekspresyon analizleri ile teyit edilmıştir. Spontan farklılaşma potansiyelini doğrulamak için in vitro embriyonik cisimcik (EC) oluşum deneyi yapılmıştır.

**Bulgular:**

Fibroblastlar ve MM hastalarından izole edilmiş MKH’ler; dört Yamanaka faktörü olan Oct3/4, Sox2, Klf4 ve c-Myc ile yeniden programlama vektörleri içeren Sendai virüsü kullanılarak yeniden programlanmıştır. İlk olarak, mikroskobik analiz ile üretilen uPKH’lerin klasik embriyonik kök hücre (EKH) benzeri morfolojik özelliklere sahip olduğu ortaya konmuştur. İkinci olarak, her iki hücre hattının da pluripotent aşamaya kadar yeniden programlanabildiği akış sitometrisi, IF boyama ve gen ekspresyon analizleri ile teyit edilmiştir. İn vitro embriyonik cisimcik (EC) oluşum deneyleri ile spontan farklılaşma potansiyeli gösterilmiştir.

**Sonuç:**

uPKH’ler MM hastalarından ilk kez başarıyla elde edilmiştir ve bu hücrelerin MM hastalığının arkasındaki moleküler mekanizmaları netleştirebileceği düşünülmektedir.

## Introduction

Yamanaka and Takahashi made a discovery in the world of life science by transforming mouse somatic fibroblasts into pluripotent cells as a result of transferring 4 gene sets (*Sox2*, *Oct4*, *Klf4*, and *c-Myc*) in 2006 [1]. Since that day, induced pluripotent stem cells (iPSCs) are considered to be one of the main sources for regenerative medicine, similarly to embryonic stem cells (ESCs). Because of their pluripotent features, both cell types are building blocks of regenerative medicine. However, in contrast to ESCs, there are no ethical limitations or immunological problems when using iPSCs [2]. Additionally, iPSCs with disease genotypes have been used for human disease modeling [3].

To date, iPSCs have been generated from many different sources [1,4,5,6,7,8,9], including mesenchymal stem cells (MSCs). MSCs were shown to be more efficient in reprogramming compared to other somatic cells [10,11].

In the last decade, iPSCs have proven to be a powerful in vitro system for studying diseases [12,13], especially genetic disorders [1,14]. Patient-specific iPSCs have powerful potential in regenerative medicine and notably in human disease modeling [15]. The in vitro phenotype of disease-specific iPSC-derived cells can enable us to comprehend the differences and/or similarities between molecular/cellular pathophysiology and clinical phenotype. This technology can also facilitate and improve the understanding of disease pathology. To date, many disease models have been established with iPSCs. There are efforts for drug screening tests and genetic modifications of cells for the treatment of diseases [15]. On the other hand, in many diseases, patient-specific iPSCs have been shown to exhibit the characteristics of the diseases [13,16].

The use of iPSCs is also very important in research on the cancer microenvironment [17,18,19]. Multiple myeloma (MM) progresses with the uncontrolled increase and accumulation of malignant plasma cells in the bone marrow (BM) [20]. MM bone disease is observed due to the increase of osteoclastic activity via the factors synthesized from malignant plasma cells and the decrease in the differentiation of osteoblasts originating from MSCs. Imbalance in this process leads to overproduction of the many responsible chemokines and cytokines and various signaling cascades are also involved in this complex process [21,22,23]. Advanced lesions and fractures occur as a result of this imbalance in bone formation and destruction.

The construction and differentiation of osteoblasts from MSCs is controlled by many factors and pathways in the BM microenvironment. Various inhibitory substances released by plasma cells in the BM microenvironment in MM stop bone formation as a result of disruption of different stages of osteoblastogenesis [24,25,26]. Furthermore, there are many factors in MM disease that disrupt osteoblastogenesis with different pathways. Many of these factors may be indirectly secreted or secreted by MM cells [27,28]. In previous studies, osteogenic differentiation defects were detected in BM-derived MSCs (BM-MSCs) obtained from MM patients, even in vitro, where MM cells did not have all the inhibitory factors secreted [29,30,31,32,33].

In recent years, various approaches have been developed in the treatment of MM bone disease, especially regarding the use of MSCs [34,35]. However, the limited proliferation of BM-MSCs obtained from MM patients and the low capacity of osteoblastic differentiation under in vivo and in vitro conditions will prevent possible autologous MSC treatments in the future. In addition to the purpose of revealing the molecular development stages of diseases and helping to design disease-specific or personalized drugs, iPSC technology is expected to show potential for future use in cell therapy or tissue engineering in many disease models. With the development of iPSC technology, it will be possible in the future to obtain genetically repaired autologous stem cells from patients or reproduce and replace the missing tissue.

Recently, different types of cells collected from patients with various diseases have been used for generating iPSCs, but this has not included patients with MM. Based on all this information, we aimed to obtain the MM disease model for the first time by reprogramming BM-MSCs obtained from MM patients in our study. Such iPSCs have serious potential to begin with because of their MM patient cell origin and the inclusion of disease genotype in a stem cell. MM-iPSCs would undeniably contain the genotype that causes the disease. With this study, patient-specific cells will make patient-specific disease modeling possible, and defects in MSCs can be studied by programming them into the pluripotent stage. This research will lead to other studies being carried out for the first time in the literature.

## Materials and Methods

### Selection of Patients and Control Groups

In this study, MSCs were isolated from BM obtained from the iliac crest of newly diagnosed MM patients (n=3). Biopsies were performed for diagnosis, staging, and evaluation of ongoing treatment.

Control samples to generate iPSCs were derived from newborn babies’ foreskin fibroblasts after obtaining informed consent approved under standard protocols.

### MSC Isolation from MM Patients and Cell Culture

The isolation and culturing of human BM-MSCs were performed as previously described by Karaöz et al. [36]. Briefly, BM aspirates were obtained from the iliac crest of MM patients. Samples were then diluted to 1:3 with phosphate-buffered saline (PBS). Histopaque-1077 (1.0777 g/mL; Sigma-Aldrich, St. Louis, MO, USA) was used for gradient centrifugation. Low-density mononuclear cells were collected and plated in tissue culture flasks.

### iPSC Generation

**Figure 1:**
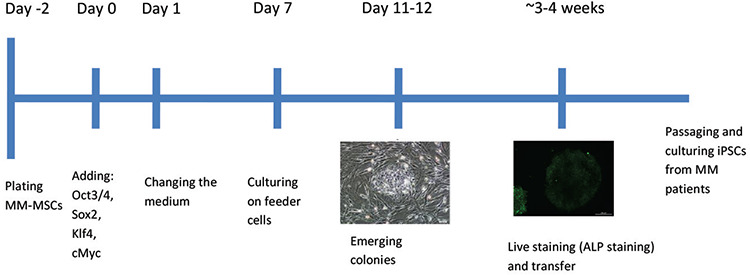
Experimental timeline for the reprogramming experiment for MM-MSCs.

For the generation of iPSCs, the CytoTune-iPS Reprogramming Kit (Thermo Fisher Scientific, Waltham, MA, USA) was used. The manufacturer’s instructions were followed for setting up the generation procedure (Figure 1). Two days before transduction, the cells were plated into 2 wells of a 6-well plate (day -2). On the day of transduction (day 0), cell medium was aspired and Yamanaka factors were added to cells, which were then incubated overnight. The cells were then cultured with their specific culture media for 6 days. When the colonies had grown to an appropriate size for transferring, live staining was done using alkaline phosphatase (ALP Live Stain, Thermo Fisher) for selecting reprogrammed colonies. The selected colonies were then harvested. Manually picked colonies were transferred onto fresh MEF plates. The next day, the medium was changed to iPSC medium (DMEM-F12 + 20% KnockOut Serum Replacement, 100 µM MEM non-essential amino acids, 1x GlutaMAX, 100 µM β-mercaptoethanol, 0.2% Primocin, and 4 ng/mL FGF) and was replaced everyday thereafter. Colony formation was monitored and photographed every day.

### iPSC Culture

iPSCs were passaged to avoid overgrowth and to maintain them in an undifferentiated state. Before splitting the colonies, differentiated colonies were removed under a microscope in sterile conditions. Differentiated areas were excised and discarded before bulk passaging. Colonies were mechanically cut into pieces using a needle for passaging. Colonies were usually ready to be passaged in 2-3 days.

For feeder-free culture, picked colonies were added to freshly prepared plates coated with Geltrex™ (Invitrogen, Life Tech., Carlsbad, CA, USA). The medium was gradually changed to StemPro^®^ hESC serum-free medium (Invitrogen, Life Tech.) as explained in Table 1. StemPro^®^ was used every day thereafter. The colonies were passaged at a 1:3 ratio. Continued passaging was done with the StemPro® EZPassage™ Disposable Stem Cell Passaging Tool (Invitrogen, Life Tech.).

### Characterization of iPSCs

### Cell Staining

The same method used for immunofluorescence (IF) staining of MSCs (Supplemental Materials and Methods) was applied. The following primary antibodies were used for staining: Oct4, NANOG, TRA1-60, TRA1-81, and Sox2 (Table 2). DAPI was used for nuclear staining.

### Flow Cytometry

The expressions of pluripotency-associated markers were analyzed by flow cytometry. Feeder-free cultured iPSCs were passaged by TrypLE (Life Technologies, Waltham, MA, USA) to be prepared as a suspension. The cells were stained with antibodies for SSEA4, Tra1-81, and Oct3/4 (BD Biosciences Pharmingen, San Diego, CA, USA).

### Gene Expression Analysis

Cell-specific gene expressions *(Lin28*, *Nr6A*, *Klf4*, *FoxD3*, *Myc*, *Utf1*, *Msx1*, *Gata6*, endogenous *Oct4*, endogenous *Sox2*, *Nanog*, and *Rex1*) in the undifferentiated cells were determined by PCR as previously described [37]. Gene expression level detection was done with LightCycler 480 DNA SYBR Green I Master (Roche, Mannheim, Germany) with specific primers on a LightCycler 480 real-time PCR instrument (Roche). The PCR reactions performed for *GAPDH* (reference gene) were as follows: 45 cycles of denaturation, 10 s at 95 °C; annealing and extension, 30 s at 60 °C. Analysis of the results was performed using Roche LightCycler 480 software.

### In Vitro Embryonic Body Formation

Spontaneous embryonic body (EB) generation was used for testing the in vitro differentiation capacity of iPSCs. Cells were cultured with medium without bFGF2 in bacteriological culture dishes for 21 days. Formation was monitored daily.

## Results

### iPSC Generation and Culture

**Figure 2:**
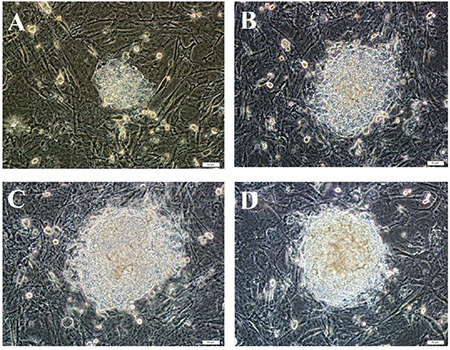
Development of the first iPSC colonies produced after Sendai virus transfection was monitored. A) On the 6^th^ day after transfection, B) 7^th^ day after transfection, C) 8^th^ day after transfection, and D) 9^th^ day after transfection. Scale bars: 50 μm (A, B, C, and D). iPSC: Induced pluripotent stem cell.

The first colonies were obtained on the 6^th^ day of culture after transduction. The structure of these colonies had a scattered appearance compared to ESC colonies, but the boundaries became more apparent in the following days. After the colonies reached a certain size, they were transferred to new feeder cell layers by mechanical passaging. These new colonies were observed to form tight cell assemblies with clearly defined boundaries observed as ESCs. Colony-like structures were photographed under the microscope as they grew over the days (Figure 2). When differentiated parts were identified, those parts were cleaned and the culture was continued. During culturing, the colonies kept their borders.

**Figure 3:**
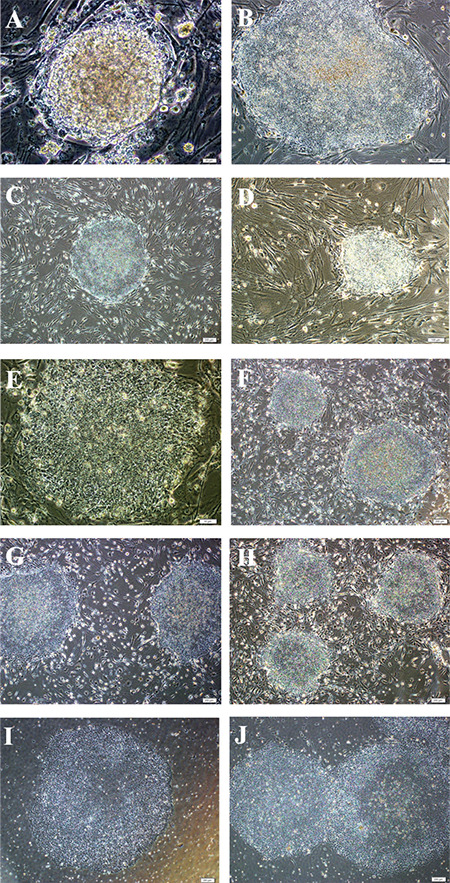
Development of new iPSC colonies obtained by mechanical passaging method was observed in culture plates. A, B, C, D, E, F, G, and H) iPSC colonies cultured in the feeder layer were picked up and cultured under feeder-free conditions. I and J) Microscopic views of iPSC colonies in the culture plate, not feeder free, are monitored. A) 2^nd^ day of P0, B) 14^th^ day of P0, C) 3^rd^ day of P1, D)4^th^ of P1, E) 2^nd^ day of P2, F) 5^th^ day of P2, G) 5^th^ day of P3, H) 7^th^ day of P3, I) 2^nd^ day of P0 on Geltrex, J) 5^th^ day of P0 on Geltrex. Scale bars: 20 μm (A), 100 μm (B, D), 200 μm (C, F, G, H, I, and J), 50 μm (E). iPSC: Induced pluripotent stem cell.

Following the mechanical passaging of colonies cultured on MEF, colonies were successfully grown in Geltrex-coated culture dishes. It was observed that the colonies retained their classical morphology (Figure 3).

### Characterization

**Figure 4:**
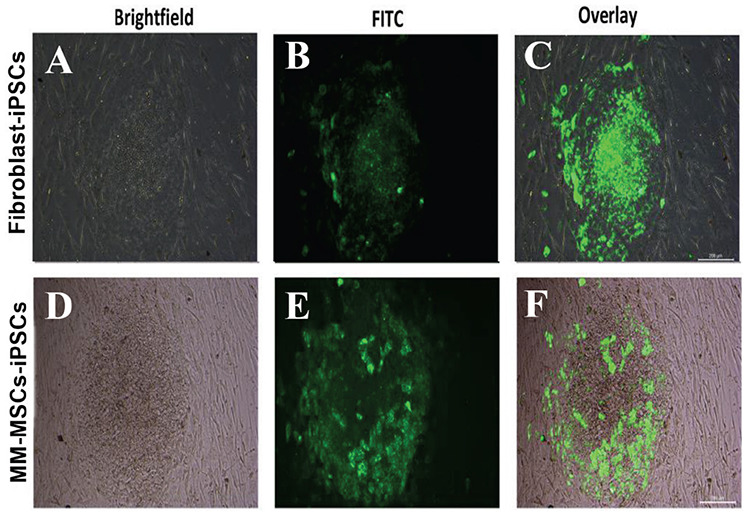
Combined images of light and fluorescent microscopes in which produced iPSC colonies reacting positively with ALP are observed. A and D) Brightfield; B and E) FITC; C and F) overlay. Scale bars: 200 μm. iPSC: Induced pluripotent stem cell; ALP: alkaline phosphatase.

The resulting colonies were stained against ALP while on the feeder layer in culture dishes. Colonies were marked with ALP-FITC dye without loss of viability. With this labeling, cells in colonies with ESC characteristics were stained (Figure 4). Green colonies were selected under fluorescence microscopy and the first cell lines were formed by physical passaging.

**Figure 5:**
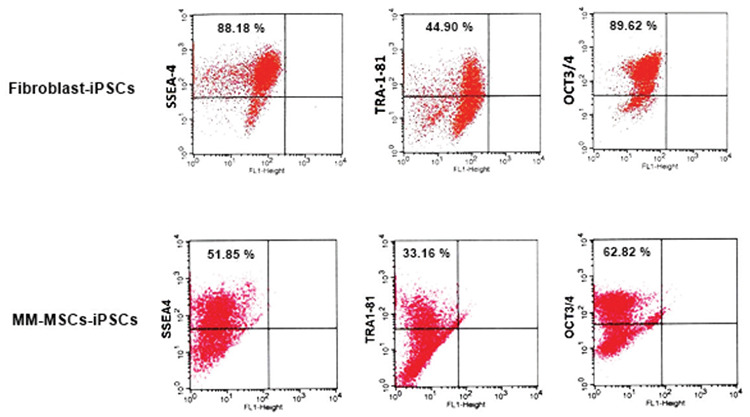
Flow cytometric analysis of pluripotency marker antigens (SSEA-4, Tra-1-81, and Oct 3/4) in normal fibroblast iPSCs and MM-MSCs-iPSCs. iPSC: Induced pluripotent stem cell.

**Figure 6:**
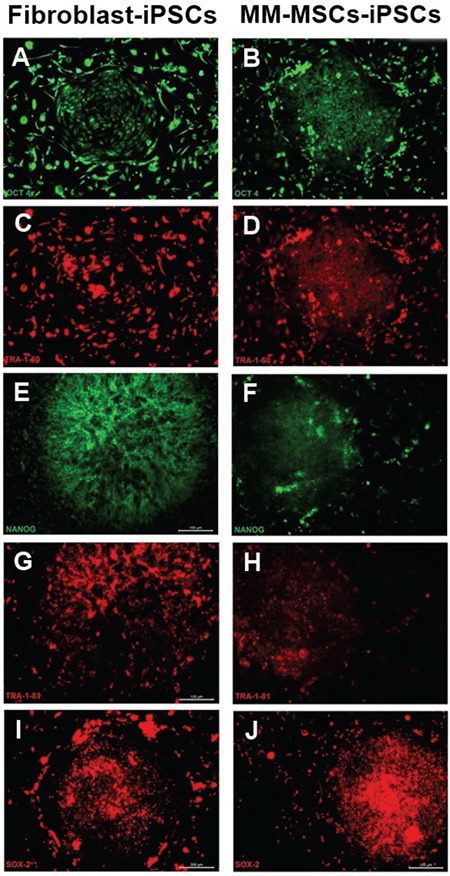
Immunofluorescence staining of pluripotency marker antigens Oct4 (A, B; green), TRA1-60 (C, D; red), Nanog (E, F; green), TRA1-81 (G, H; red) and Sox2 (I, J; red) in fibroblasts and MM-MSCs-iPSCs. All markers were positive for the colonies. Scale bars: 20 μm (A), 100 μm (B, D), 200 μm (C, F, G, H, I, and J), 50 μm (E).

ESC markers such as SSEA-4, TRA-1-81, and Oct3/4 were positive for cells in flow cytometry analysis (Figure 5). iPSCs cultured on feeder layers were further characterized by IF methods. The colonies were positive for pluripotent cell markers Oct4, TRA1-60, Nanog, TRA1-81, and Sox2 (Figure 6).

**Figure 7:**
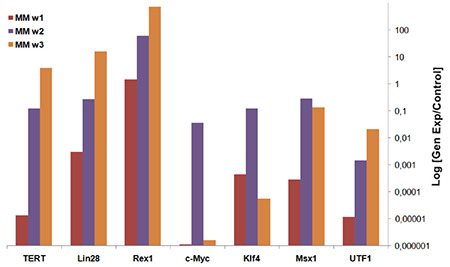
Pluripotent gene expression analysis of colonies formed after viral infection. Gene expressions were monitored for 3 weeks (w1, w2, w3). As a result, it was seen that iPSCs express pluripotent markers.

According to expression analysis, a significant increase was observed between the 1^st^ and 3^rd^ weeks for iPSC cultures. The significant increase in *c-Myc* and *Klf4* gene expressions in the 2^nd^ week decreased in the 3^rd^ week. Since these genes are transmitted by viruses, the initial expression was ectopic and turned into the internal expression of the cells at the 3^rd^ week (Figure 7).

**Figure 8:**
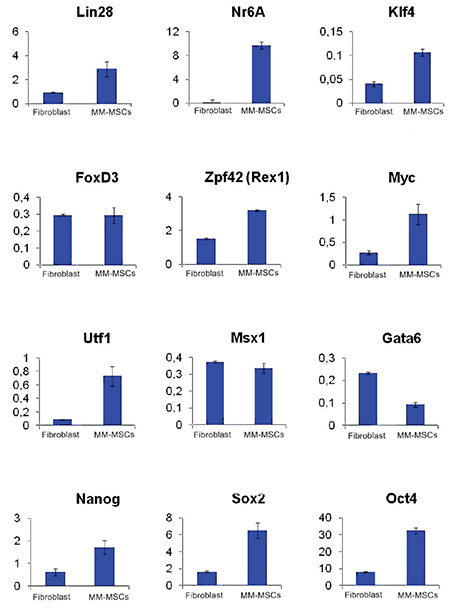
Measurement of the expression of pluripotent genes by real-time polymerase chain reaction. The *HPRT* gene was used as the reference gene. Gene expression values are expressed according to fold values relative to the *HPRT* gene. iPSCs obtained from fibroblasts were used as a control in gene expression analysis.

**Figure 9:**
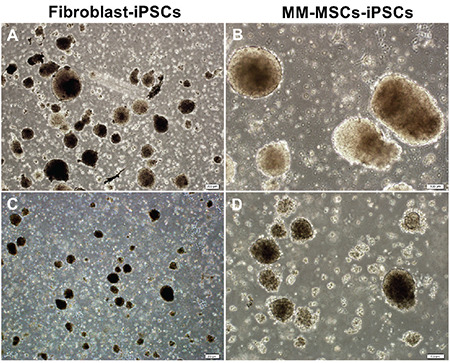
Embryoid body (EB) formation of iPSCs. A, B) EBs generated from fibroblasts. C, D) EBs generated from MM-MSCs. Scale bars: 200 μm (A and C), 100 μm (B and D).

Expressions of pluripotent genes were shown in all obtained iPSCs. Significant increases were observed in the *Oct4*, *Nanog*, *Sox2*, *Rex1*, *Utf1*, and *Lin28* genes. Using the Sendai virus, the *Oct4*, *Sox2*, *c-Myc*, and *Klf4 *genes were transferred and their expressions were provided with the help of ectopic vectors. According to these transferred vectors, the *Oct4*, *Sox2*, *c-Myc*, and *Klf4* expressions may not be the endogenous gene expressions of the cells. However, the increased expression of highly specific pluripotent genes such as *Nanog*, *Lin28*, and *Utf1* constitutes the most serious evidence that cells acquire a pluripotent cell character (Figure 8). EB formation was obtained after the 4^th^ day of suspended culturing (Figure 9).

## Discussion

iPSCs carry immense potential for future cellular therapies. However, they were also shown to carry the characteristics of the cells they originated from and their niche [38] through their epigenetic memory [39,40]. The first type of reprogrammed cells was found to be fibroblasts. Other types of human cells have also been tried for reprogramming, which might be potentially easier [40].

In this study, we attempted the reprogramming of MSCs obtained from MM patients’ BM. Our study uses a standardized reprogramming approach to evaluate the reprogramming of two cell lines in various stages of differentiation: terminally differentiated fibroblasts as a control and multipotent MSCs obtained from MM patients. Both types of cells were reprogrammed with the CytoTune-iPS 2.0 Sendai Reprogramming Kit that contains Yamanaka factors. Yamanaka factors have been reported many times in the literature as adequate for effective reprogramming [4,41,42,43]. The efficiency of iPSC generation using the Sendai virus is much higher than that of conventional vectors [43]. The elimination of the Sendai virus is also easier than that of conventional vectors, which allows the obtaining of transgene-free iPSCs.

First of all, microscopic analysis revealed that the generated iPSCs possessed classical ESC-like morphological characteristics. Secondly, reprogramming experiments demonstrated that both cell lines can be reprogrammed up to the pluripotent stage, which was confirmed by flow cytometry, IF staining, and gene expression analyses. To confirm the spontaneous differentiation potential, an in vitro EB formation assay was performed.

iPSCs have been successfully obtained from MM patients for the first time here. One of the major findings of our study is the rapid reprogramming of MSCs, which started as early as the 6^th^ day with the appearance of the first colony-forming cell accumulations. Considering the results of previous studies, this rapid reprogramming can be attributed to the multipotent nature of MSCs, which implies that the effectiveness of reprogramming is related to the differentiation stage of the cell line. Adegani et al. [44] demonstrated that human MSCs of various sources such as adipose tissue and BM-MSCs intrinsically expressed core pluripotency factors such as Lin28, Klf4, and Sox2 at higher levels with Nanog at moderate levels and Oct4 at low levels, which allows them to reprogram easily.

Our data show that human iPSCs can be derived from MSCs more rapidly than fibroblasts. Obtaining MSCs from patients does not require great effort because BM aspirates are taken almost daily for diagnostic purposes in hematology clinics. MSCs can be isolated from these samples. As a result, we generated iPSCs from MM-MSCs for the first time. As we know from previous studies, the osteogenic differentiation of MM-MSCs is weak. Our next goal is to explore the differences between the osteogenic differentiation potential of healthy donors’ MSCs-iPSCs and MM-MSCs-iPSCs. We are planning further studies to understand the pathogenesis of this disease, because MM-iPSCs could clarify the molecular mechanisms behind the disease. Therefore, further studies should be developed to understand the molecular mechanisms of this disease. Understanding the pathogenetic mechanisms underlying the disease is crucial for effective management and improving the quality of MM patients’ lives [22]. Based on current knowledge, the investigation of novel targeted drugs and an understanding of the role of novel targeted therapies in this disease are of great interest [23]. For more successful results, researchers are developing complex 3D environments using MM patients’ cells [45]. iPSCs offer unprecedented opportunities for drug discovery and screening with their ability to differentiate into all kinds of cells found in the body.

## Conclusion

The data obtained from this study confirm that iPSCs can be derived from MSCs more rapidly than fibroblasts and iPSCs have been successfully obtained here from MM patients for the first time. iPSCs generated from MM-MSCs could clarify the molecular mechanisms behind this disease. Thus, further studies should be developed to understand the molecular mechanisms of this disease. Our next goal is to discuss the differences between the osteogenic differentiation potential of healthy donors’ MSCs-iPSCs and MM-MSCs-iPSCs.

## Supplemental Materials and Methods

### Fibroblast Isolation

Foreskin samples were obtained from circumcision procedures under sterile conditions and fibroblasts were derived using a previously described culture method [46].

### Characterization of MSCs

To confirm the phenotypic characteristics in vitro, MSCs at passage 3 (P3) were analyzed. For the characterization, flow cytometry analysis, IF staining, and differentiation studies were performed.

### Flow Cytometry

To confirm that MM-MSCs maintain their phenotypic characteristics in vitro, undifferentiated MSCs were analyzed by flow cytometry. Analyses were performed on a FACSCalibur (Becton Dickinson, San Jose, CA, USA) with Cell Quest software (BD Biosciences, Bedford, MA, USA). MM-MSCs were immunophenotyped with antibodies against human antigens (CD45, CD59, CD14, CD117, CD11b, CD34, CD44, CD90, CD15, CD33, CD105, CD73, CD29, CD38, CD138, and CD166), as well as their isotype controls immunglobulin G [(IgG1), (IgG1/G2a)](BD Biosciences).

### Immunofluorescence Staining

For cellular marker identification, cells at P3 were seeded onto poly-L-lysine-coated 8-well chamber slides (BD Biosciences). Cells were cultured for another 1-2 days and then stained. For the determination of the expressed protein profiles, IF staining was performed with fluorescence dye-attached antibodies. IF analyses were performed as previously described [47]. Briefly, samples were rinsed in PBS and then fixed. Triton X-100 (0.025%; Merck, Darmstadt, Germany) was used for permeabilization and cells were incubated for 30 min with blocking serum (Santa Cruz Biotechnology, Heidelberg, Germany) in PBS at 37 °C to suppress nonspecific binding of IgGs. Following washing, the primary antibodies (α-smooth muscle actin, CD29, vimentin, nestin, CD34, CD44, fibronectin, vinculin, tenascin) were used for incubating the cells overnight at 4 °C. The next day, samples were incubated with secondary antibodies for 25 min at room temperature. After the washing steps, the cells were mounted with DAPI (Santa Cruz Biotechnology). Samples were examined under a fluorescence microscope (Leica DMI 4000, Leica Microsystems, Wetzlar, Germany).

## Figures and Tables

**Table 1:**
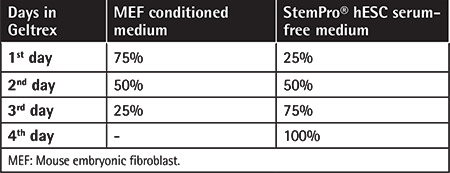
Media percentages of MEF conditioned medium and StemPro^®^ hESC serum-free medium in the first days of feeder-free culture.

**Table 2:**
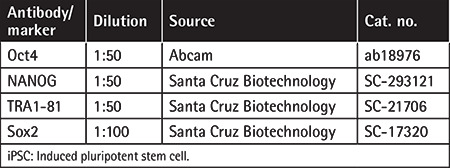
Primary antibodies used for characterization of iPSCs.
